# Observation Oriented Modeling: Principles and Tools for Person-Oriented Research

**DOI:** 10.17505/jpor.2026.29450

**Published:** 2026-08-24

**Authors:** James W. Grice, Saige L. Schmidt, Emery K. Thackerson

**Affiliations:** Oklahoma State University

**Keywords:** Observation oriented modeling, person-centered, person-oriented, ecological fallacy

## Abstract

Observation Oriented Modeling (OOM) is a person-oriented approach to data conceptualization and analysis that departs significantly from traditional methodological and statistical frameworks. Rather than relying on means, variances, and covariances, OOM focuses on the patterns formed by individual observations. Instead of estimating population parameters, OOM prioritizes the development of explanatory theories based on those observed patterns, reflecting a broader philosophical shift from logical positivism toward philosophical realism. In this paper, we illustrate the principles and methods of OOM through a series of re-analyses of published studies. These examples demonstrate how OOM can reveal meaningful behavioral patterns at the individual level that are often obscured by conventional aggregate analyses. In addition to being simple and intuitive to use, OOM encourages researchers to engage more deeply with causal models and less with statistical procedures. We conclude by discussing the broader implications of OOM for person-oriented psychological science and the field as a whole.

Consider the following statements from philosophers of science, which highlight the importance of patterns in scientific investigation:

Scientific inquiry often depends on the extraction of patterns from data. (Suñé & Martínez, 2021, p. 4315)

Theories are seen as solutions to a peculiar style of problem: namely, “Why is it that the patterns of phenomena are the way they are?” A theory answers this question by supplying an account of the constitution and behavior of those things whose interactions with each other are responsible for the manifested patterns of behavior. (Harré, 1986, p. 35)

What are best termed the “abstract sciences” aim at an understanding of the fundamental processes of nature. Such inquiry may be motivated by discerning a pattern, but not all patterns will be of concern. Indeed, patterns which emerge from experimentally generated data, e.g., the results of Lavoisier’s painstaking use of the chemical balance, are of high importance. (Manicas, 2006, p. 25)

Taken together, these quotes indicate that, in gaining scientific knowledge about the world, a researcher does not merely accumulate facts but instead engages in a process of detecting and causally explaining meaningful patterns in nature. This process can take on a decidedly quantitative flavor; for example, when Gregor Mendel observed consistent inheritance ratios in pea plant traits and explained them through the action of discrete hereditary units. It can also take on a qualitative flavor, as when August Kekulé discovered the molecular structure of benzene. The empirical formula for benzene was known at the time, but it was Kekulé’s identification of its structure (i.e., its *pattern*, six carbon atoms connected in a symmetrical arrangement with alternating single and double bonds) that opened the door to the science of aromatic compounds.

In the domain of psychology, regarding science as pattern detection and explanation becomes particular *à propos* in person-oriented (or person-centered) research, where psychologists are asked to shift their focus from summarizing aggregates and estimating population parameters to studying individuals and developing causal models that explain behavior. In their paper, *A Person-Oriented Approach: Methods for Today and Methods for Tomorrow*, Bergman and ElKhouri note:

To sum up, from a person-oriented perspective within a holistic-interactionist paradigm, a person-oriented approach based on the study of *patterns of values* in relevant variables appears to be useful in a number of settings…A typical *value pattern* may not appear with the same frequency under varying conditions, but as long as the basic system design is the same, we can expect the same *typical patterns* to emerge again and again. (Bergman & El-Khouri, 2003, p. 26, italics added)

In concert with this emphasis on patterns, Observation Oriented Modeling (OOM) was developed for psychologists as an alternative approach to both data conceptualization and analysis (Grice, 2011, 2014). In the present paper we provide an updated overview of the data-analytic tools available in OOM by demonstrating their application to a series of empirical data sets. Through these examples we illustrate central features of the OOM perspective: (1) the use of visual displays and relatively simple mathematical operations rather than formalized systems of equations, (2) a focus on patterns of observations at the level of the individual, (3) the assessment of explanatory plausibility through case-level matching rather than aggregate parameter estimation, and (4) the construction and evaluation of causal models informed by Aristotle’s rich conception of causality, in which explanations are understood as accounts of the structures and processes that give rise to the observed patterns in the data.

With each example we will first describe the data, then consider the relevant causal processes, and finally demonstrate how the data are analyzed within the OOM framework. Because Aristotle’s conception of causality will play a central role in the following examples, a brief overview of his four causes (material, formal, efficient, and final) is warranted. Consider, for example, the experience of depression. We may approach depression in terms of *material* causes by pointing to neurochemical processes, for example, those involving neurotransmitters like dopamine or serotonin. Here the focus is on the material constituents underlying the depressive condition. Depression may also be understood in terms of *formal* causes. From this perspective, depression is not reducible to isolated symptoms or biological components, but instead consists of an organized pattern or structure of thoughts, emotions, and behaviors. A different configuration of these same elements might instead constitute anxiety or some other psychological condition. Formal causes therefore concern the pattern, organization, or form that makes a thing the kind of thing it is. Attention may also be directed toward *efficient* causes, that is, the processes or events that bring about or alter the depressive state such as the dissolution of a romantic relationship, the death of a loved one, chronic stress, or failure on an important examination. Efficient causes thus concern the processes of generation, change or development over time. Finally, depression can be considered in terms of *final* causes, which involve ends, goals, or purposes. A person may become or remain depressed because he or she cannot envision a hopeful future, because the condition serves some protective or interpersonal function, or because it has become integrated into the person’s broader orientation toward life. Final causes therefore concern the aims, purposes, or end states toward which psychological processes are directed.

Taken together, Aristotle’s four causes provide a rich framework for scientific explanation by illuminating different but complementary aspects of causal understanding. As the examples below will demonstrate, integrating this philosophical perspective with the methods of OOM entails a broader Gestalt shift in research practice that moves from “from means and variances to persons and patterns” (Grice, 2015).

## Motivating Example, Small-*n* Design

As an initial demonstration of the OOM approach, including its key terms, core ideas, and philosophical premises, we begin with a small-*n* design reported by Swan and Pustejovsky (2018). In their paper, they present data from a study conducted by Thorne and Kamps (2008) in which an ABAB research design was used to evaluate the efficacy of a low-cost, reward-based group contingency program to reduce daily problem behaviors in twelve students who were at risk for developing behavioral disorders. The primary outcome was the frequency of inappropriate behaviors, measured through direct observation of each of the twelve students. Up to four days of observations were collected during the initial baseline, up to sixteen during the initial treatment, up to seven during the second baseline, and up to fourteen during the final treatment.

**Figure 1:**
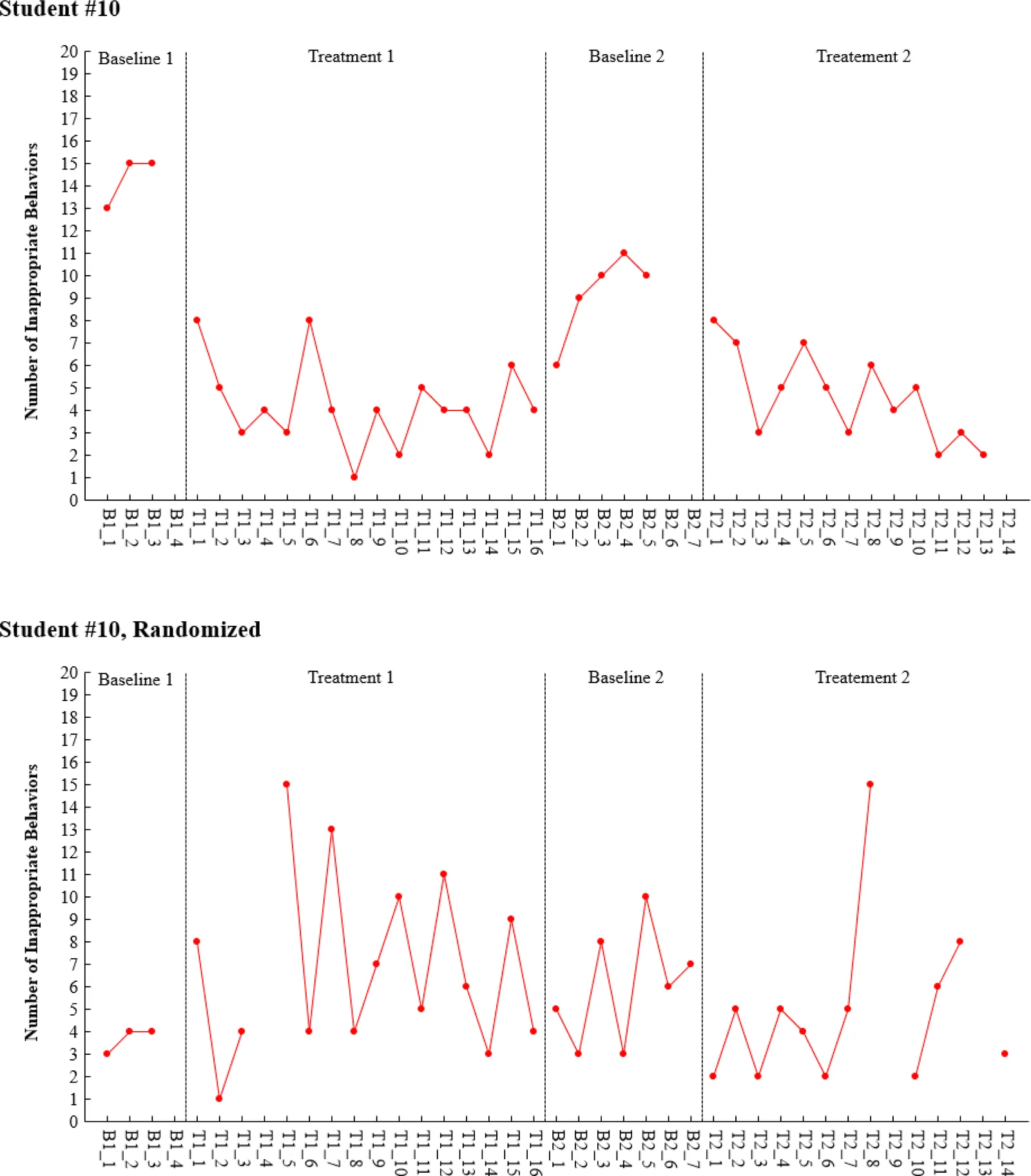
Efficient Cause Blocked Orderings results for Student #10

The top image of Figure 1, produced by the *Efficient Cause Blocked Orderings* analysis in the OOM software, shows results for Student #10. In OOM, variables are referred to as “orderings” (Grice, 2011, p. 9), and ABAB designs are commonly used to identify efficient causes. The analysis is named accordingly, and as can be seen in the figure, Student #10 began with ~14 daily inappropriate behaviors at the first baseline and then improved to ~4 daily behaviors during the first treatment block. The inappropriate behaviors then increased when the treatment was removed (Baseline 2) and finally decreased again when the treatment was reinstated (Treatment 2). This pattern offers clear, visual evidence for the efficacy of the program for this student.

This impressive pattern can be supported by the computation of a Percent Correct Classifications (PCC) index, which is the primary statistic produced by most OOM analyses. In common statistical parlance it can be considered as an index of “effect size” (Kirk, 2014; Rosnow & Rosenthal, 2003; Stegenga, 2015) that ranges in value from 0 to 100. As its name suggests, the PCC indicates the percentage of observations (often, people) that match the theoretically informed pattern under investigation. Considering Student #10 in Figure 1, the PCC is computed by tallying the pairs of observations between the adjacent baseline and treatment conditions that are consistent with the expected ordinal pattern, Baseline > Treatment, and then converting the sum to a percentage (see Gyunn & Grice, 2023; Parker & Vannest, 2009; Parker et al., 2007). With 193 comparisons, the resulting PCC is impressively high at 96.37% and supports the efficacy of the treatment protocol. In other words, comparisons of adjacent baseline and treatment observations show that, in most instances (96.37%), fewer inappropriate behaviors were recorded during treatment.

This PCC can furthermore be evaluated using a randomization test conducted in the *Efficient Cause Blocked Orderings* procedure. There are a number of ways to conduct the analysis (see Grice, 2026), but the generally preferred way is to randomly shuffle the observations within each student. The bottom image of Figure 1 shows a randomized version of Student #10’s recorded inappropriate behaviors. As can be seen, the original observations have been shuffled across the baseline and treatment blocks, and in this instance the clear pattern in the top image of Figure 1 has been corrupted. The PCC for this randomized version of Student #10’s data is equal to 43.59%. The user determines the number of such randomizations, say 1000, and in each instance the resulting PCC is computed and compared to the observed PCC (96.37%). The proportion of comparisons for which the randomized PCC equals or exceeds the observed PCC is then determined and reported as a *chance*-value, or *c*-value, in OOM. With 1000 randomized trials, the result for Student #10 is found to be 0, meaning that not in a single instance did a randomized PCC equal or exceed the observed PCC. Even with 10,000 trials the maximum randomized PCC was equal to 88.00%. Given that it is possible to randomly generate the original pattern of data and obtain a PCC equal to 96.37%, it is conventional to report the *c*-value as ‘< .001’ for 1000 trials or ‘< .0001’ for 10,000 trials, respectively, for such outcomes.

The *c*-value is then used as evidence to draw an *inference to best explanation* rather than an inference to population parameters as is done with traditional tests of statistical significance (Grice, 2021; Lipton, 2004). To understand this inference, let’s return to the top image in Figure 1 which shows a pattern in the data that is consistent with expectation (i.e., Baseline > Treatment) for a large majority of pairs of observations, 96.37% to be precise. What explains this pattern? Two generic explanations can be offered:

(1) The observed pattern is primarily the result of some operating cause or causes; that is, some generative mechanism(s). For this study the causal model would be found in the behavioral theory underlying the intervention strategy used by Thorne and Kamps (2008).
(2) The pattern is the result of *accidental causes* only, which can be referred to in the singular as “physical chance” (Wuellner, 1966).

By randomly shuffling the observations we simulated physical chance; that is, we treated the order of the observations as accidental (or haphazard, if you will). If such accidental orderings lead to PCCs that are as high or higher than the observed PCC, resulting in a high *c*-value, then we can *plausibly* infer that physical chance is the best explanation of the observed pattern. If a low *c*-value is found, as in the current example, then we can *plausibly* infer that some cause or causes are operating that provide better explanations of the observed pattern. In Aristotelian-Thomistic philosophy (Wallace, 1996), upon which OOM rests, causes are treated as *explanatory principles*; accordingly, a causal interpretation constitutes an explanation of the pattern insofar as it identifies its relevant causes. It is also helpful to keep in mind that accidental causes are not *per se* causes but causes connected to effects incidentally. By contrast, *per se* causes produce their effects in accordance with the natures of the entities in which they are embodied and therefore do so necessarily; in other words, effects depend essentially upon their *per se* causes. For Student #10, the low *c*-value (< .0001) supports the plausibility of the causal interpretation of his or her pattern of inappropriate behaviors across the ABAB design.

**Figure 2:**
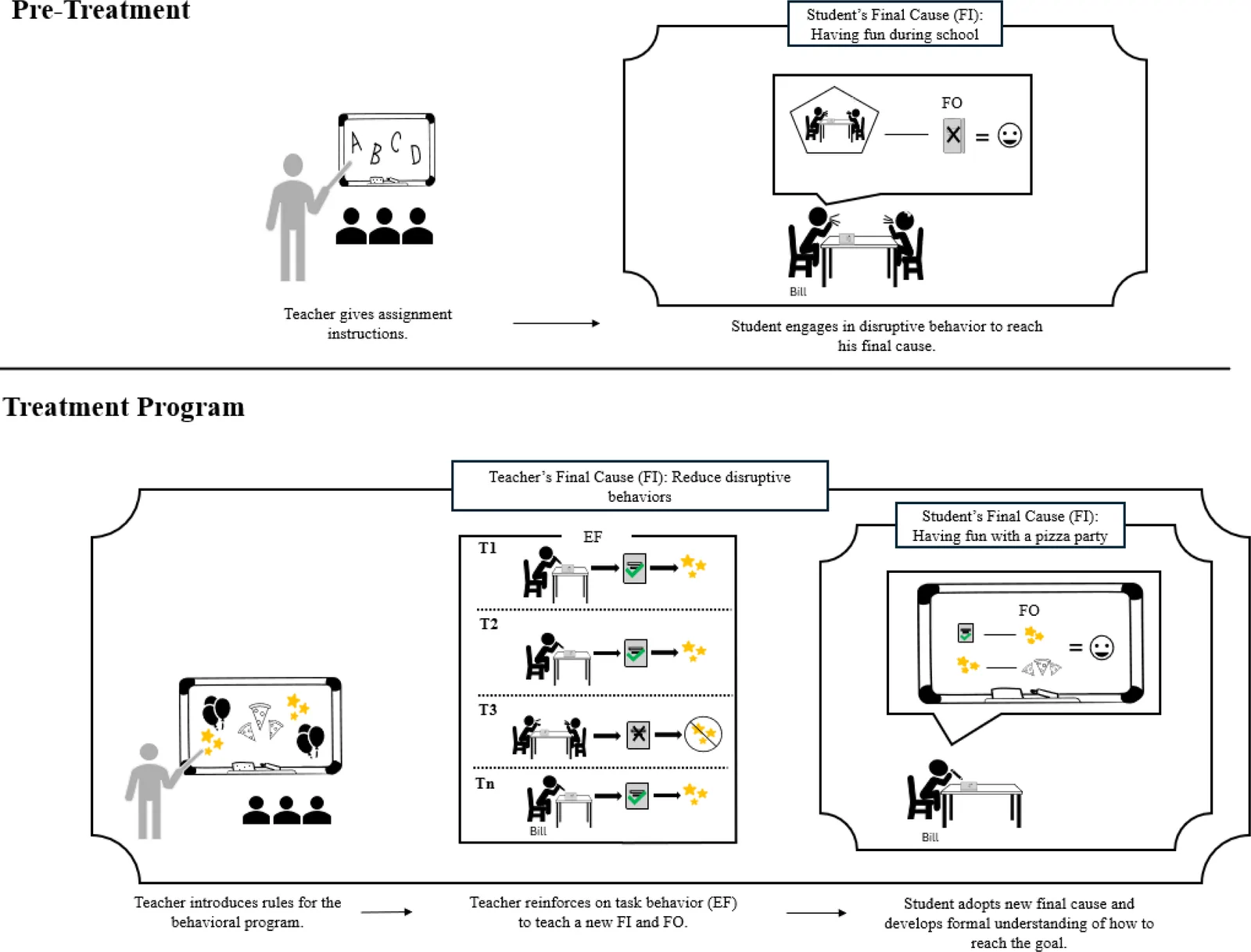
Integrated model for behavioral modification program

Considered as a whole, the teacher’s treatment program can be regarded as an efficient cause. When the treatment is present, Student #10’s disruptive behaviors are mostly replaced by appropriate behaviors; but what is the specific causal structure of the program? Figure 2 shows a candidate explanatory model in iconic form for Thorne and Kamps’ (2008) study. Ideally, such a model would be constructed as part of the study design, but we present it here for illustrative purposes. The top panel of the model shows the essential features of the pre-treatment classroom; namely, a teacher giving instruction and a student, Bill, engaging in disruptive behavior with another student. The ticket labeled “Student’s Final Cause: Having fun in class” represents Bill’s goal of having fun in class. Within the callout above Bill’s head is a pentagon which represents complex judgments he is making when engaging in specific behaviors with the other student and which the teacher regards as disruptive. There is also an important structural aspect of Bill’s internal world; namely, if he engages in socially stimulating and pleasurable disruptive behavior with another student, then he will not have to complete the assignment, which he regards as a barrier to having fun. This “if…then” logic-like relationship is a formal cause and is represented by the FO label in Bill’s callout.

The bottom panel of Figure 2 represents the treatment phase of the study. The teacher attempts to reduce disruptive behaviors by introducing a new reinforcement system. Because this is the umbrella final cause upon which everything else depends, it is represented by a large ticket that surrounds all other components of the model. She achieves her goal by establishing a new potential final cause for Bill’s behavior, the opportunity to earn rewards and a pizza party, and teaches the rules for obtaining these rewards. As can be seen in the figure, this process begins with an explanation of the reinforcement program, including how students can earn cards (represented by stars) and how those cards earn a future pizza party through the group contingency. Through repeated reinforcement of on-task behavior with reward cards (EF, for efficient causes), Bill develops a new formal understanding (FO) of how his actions can lead to the desired outcome. In other words, he learns the “rules of the game”, working on task earns cards and the accumulation of cards by himself and his classmates earns the pizza party. Because Bill values the pizza party, he begins to engage in behaviors that increase the likelihood of obtaining that reward. Working on task becomes a means of achieving this new final cause and is incompatible with disruptive behavior. As a result, when the contingency is in effect, Bill’s disruptive behavior decreases because it is incompatible with the behavior required to reach his goal. The teacher thereby reduces disruptive behavior and achieves her own final cause.

When the teacher removes the immediate rewards and pizza party during the second baseline condition, Bill reverts to his original final cause (having fun in class) and once again avoids completing assignments through disruptive behavior. The model presented in Figure 2 is therefore explicitly teleological rather than reflecting a strictly efficient-cause-only, operant conditioning account of student behavior. We discuss the rationale for this modeling choice in greater detail below, but the purpose of presenting the model here is to illustrate how an inference to best explanation can be represented in an iconic model that incorporates Aristotle’s different species of cause. The low *c*-value reported above for Student #10’s pattern of disruptive behaviors indicated that the construction of such an explanatory model is warranted.

**Figure 3:**
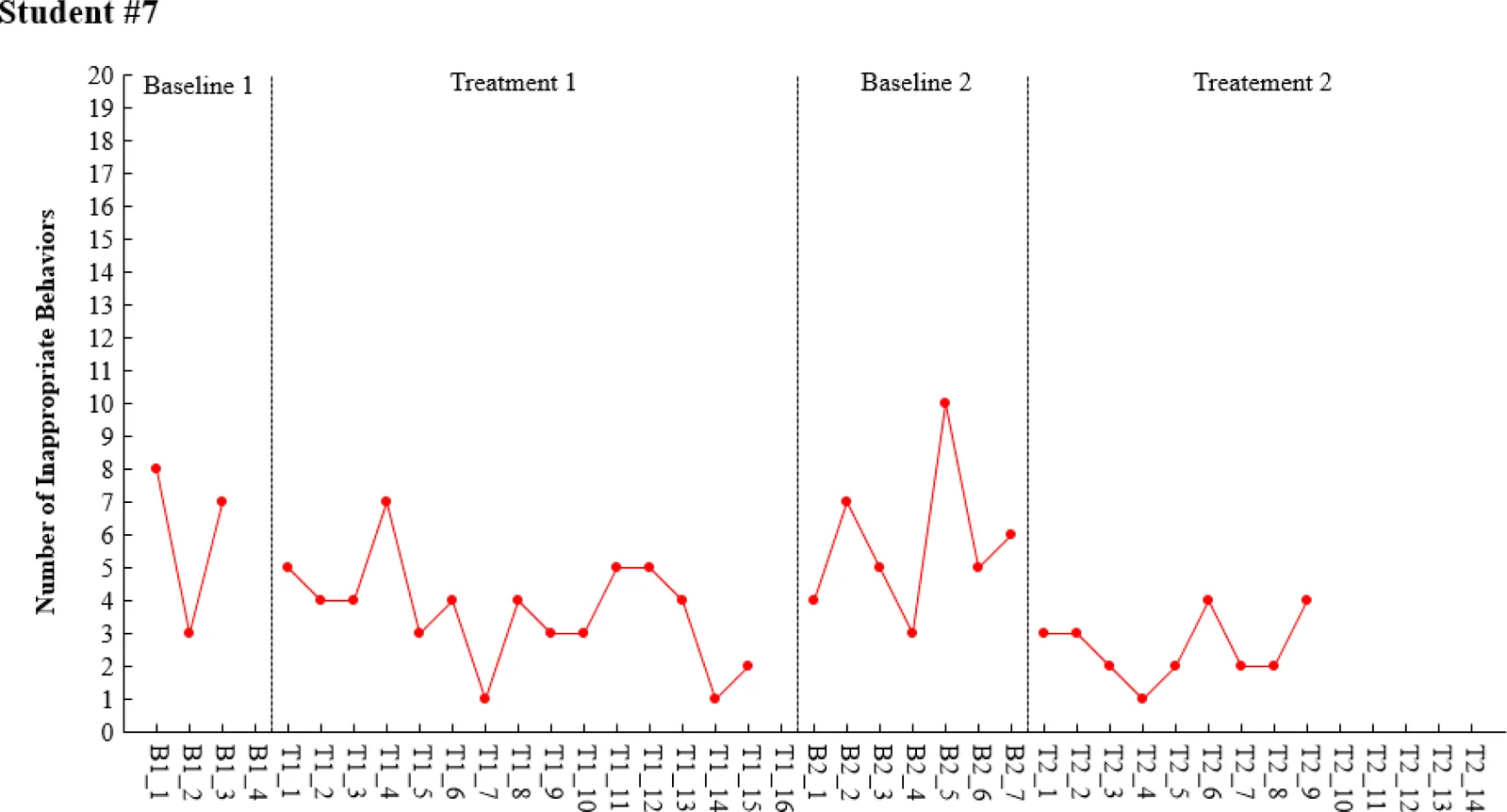
Efficient Cause Blocked Orderings results for Student #7

To conclude this motivating example, the plots for the other eleven students were examined and their respective PCCs and *c*-values computed and evaluated. Results for these students were similar to those for Student #10. The lowest PCC was found for Student #7 (76.53%) but was still fairly high and accompanied by a low *c*-value (.002). As can be seen in Figure 3, this student started with a lower baseline of inappropriate behaviors but nonetheless showed reductions in these problem behaviors in the two treatment phases of the study. The pattern of observations, high PCC value, and low *c*-value therefore still support the efficacy of the treatment for this student. Aggregating across all observations and students is also possible in the *Efficient Cause Blocked Orderings* analysis, and the results revealed an overall PCC equal to 88.87% with a low *c*-value (< .001). At the level of the individual students and in the aggregate, then, the results from the OOM analysis indicate the behavioral program was quite successful in reducing inappropriate behaviors, thereby supporting the behavioral modification theory underlying its creation. Finally, while unneeded for the OOM analyses, mean or median changes in the number of inappropriate behaviors or other descriptive statistics could also be reported for these data at the researcher’s discretion and so long as they are not regarded as estimates of population parameters. More will be said about traditional analyses, null hypothesis significance testing, and statistical assumptions in the Discussion below.

## Efficient Cause Paired Orderings

Another procedure to analyze efficient causes in the OOM software is the *Efficient Cause Paired Orderings* procedure, which can be used when observations are collected for both the putative cause and the effect. As an example, consider the study by Wilson (2008) who sought to identify individual triggers of daily stress by asking thirteen students to complete ratings of stressful events across more than 50 consecutive days. The stress ratings were made on an 11-point (0 to 10) scale. The students were also asked to rate each daily event on 24 possible thoughts or feelings (e.g., feeling defeated, feeling anxious, fear of failure) using the same 11point scale. As stated above, the goal of the study was to identify individual “triggers” (i.e., efficient causes) of stress. For one person, for example, anxiety may trigger stress, whereas for another, fear of failure may trigger stress.

**Figure 4:**
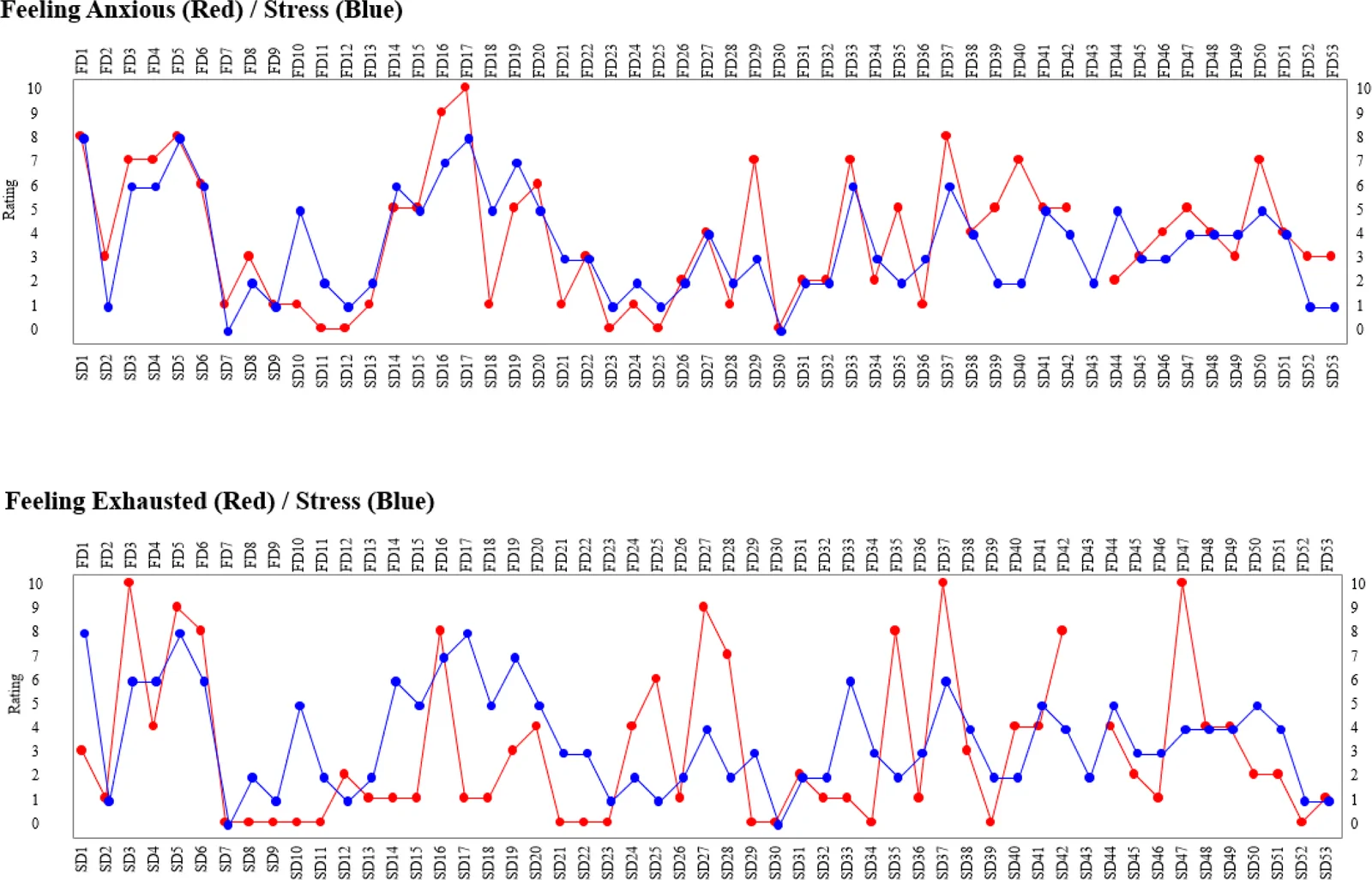
Efficient Cause Paired Orderings results for Student #9

Grice et al. (2016) showed how these data could be analyzed using the *Efficient Cause Paired Orderings* procedure which, like the blocked orderings analysis above, emphasizes visual examination of the data with the support of PCC indices and *c*-values from randomization tests. The top image in Figure 4 presents results for one participant (Student #9) for whom “Feeling Anxious” was identified as an efficient cause (i.e., a “trigger”) of daily stress. As can be seen, changes in the anxiety and stress ratings are mostly simultaneous across the 53 days. For instance, the relatively low ratings on day two are followed by relatively high ratings on day three. In other words, both anxiety and stress increased from day two to day three. A PCC index can be computed by tallying the number of instances for which simultaneous increases or decreases are observed across the pattern. With 53 days, and one missing anxiety rating on day forty-three, there are 50 changes to be tallied, and for this participant the percentage was high, 70.00% (35 / 50 * 100). Repeatedly randomizing within the stress ratings, results from a randomization test also supported a causal inference (*c*-value < .001). By way of comparison, as can be seen in the bottom image of Figure 4, ratings of “Feeling Exhausted” for this same student were not related to ratings of daily stress, PCC = 42.00%, *c*-value = .207.

**Figure 5:**
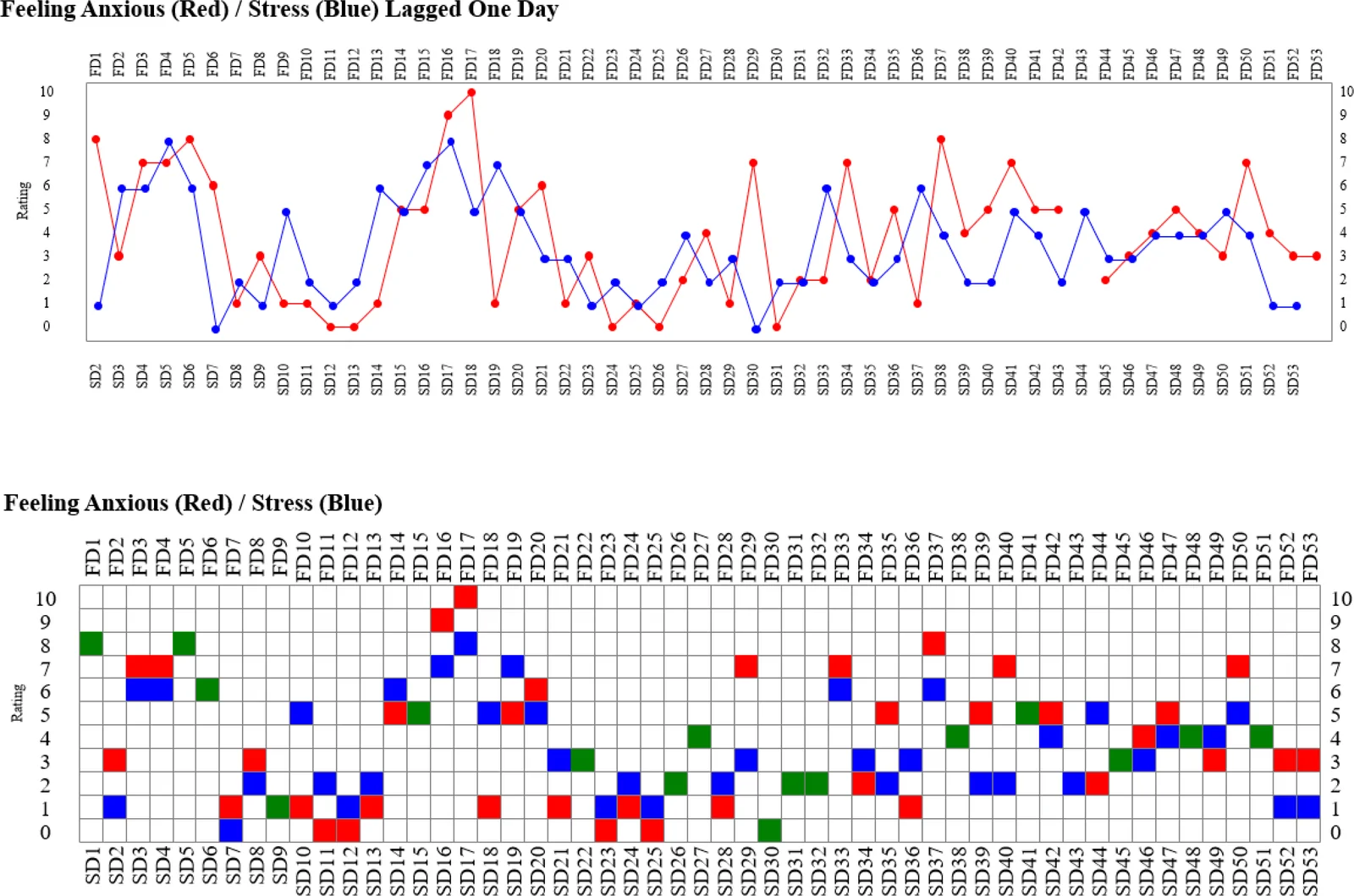
Efficient Cause Paired Orderings results for lagged and matched analyses, Student #9 *Note*. Student #9’s stress ratings have been lagged one day in the top image. Notice how SD2 (the stress rating for day 2) is aligned with FD1 (the anxiety rating for day 1), SD3 is aligned with FD2, and so on. The bottom image shows the original ratings for Student #9 in color-coded form. The green cells indicate days when the same scale value was used for both anxiety and stress.

Grice et al. (2016) visually examined each of the 13 students and identified at least one convincing trigger (i.e., efficient cause) of stress for 8 students (PCC ≥ 60%). For one student, 19 of the 24 thoughts or feelings were identified as triggers, and no clear triggers were identified for the other 5 students. The two most commonly identified triggers of stress were feelings of discouragement and feelings of being overwhelmed (each reported by 4 students). Feelings of failure, exhaustion, being behind, frustration, and anxiety were also identified as potential triggers for three students. These findings support Wilson’s (2008) original hypothesis that triggers of stress vary considerably across individuals, although it must be pointed out that efficient causes necessarily entail events ordered in time, as when billiard balls collide sequentially or when a bird first selects a location for the construction of its nest. The two sets of ratings were not linked temporally in Wilson’s study, however, and the participants were not asked whether they considered the thoughts or feelings to be causes of stress or consequences of it. This ambiguity limits the strength of the efficient cause inference that can be drawn from the study. Clear patterns, high PCCs, and low *c*-values, like the results in the top image of Figure 4, do not resolve this uncertainty. One must always turn to theory. In their analysis of Wilson’s data, Shoda, et al. (2013) drew upon the Cognitive Affective Processing System (CAPS) theory (Mischel & Shoda, 1995) to support the inference, and Grice et al. (2016) constructed an integrated model illustrating how thoughts and feelings could function as efficient causes of stress in the context of the study design. As with other procedures in the OOM software, the *Paired Orderings Efficient Cause* analysis offers numerous options that cannot be fully covered here. However, two features are particularly relevant. First, the analysis allows for testing lagged effects, similar to cross-lagged panel models and multilevel modeling. For example, the top image of Figure 5 shows Student #9’s stress ratings lagged by one day to test whether stress tends to appear the day after an anxious event. This is only one of several plausible causal hypotheses. Stress may instead precede anxiety, or the two variables may reflect different manifestations of a common underlying process or state rather than a direct causal relationship. The lagged analysis provides one means of evaluating these competing possibilities by determining whether a particular temporal ordering better accords with the observed pattern. In this case, the one-day lag substantially weakened the pattern, reducing the PCC from 70.00% to 24.49% and increasing the *c*-value to .984. Second, rather than analyzing differences across time points, pairs of responses can be strictly matched. As shown in the bottom image of Figure 5, Student #9’s ratings appear as colored cells in an 11 × 53 matrix. Green cells indicate days on which the student assigned the exact same rating to both the anxiety and stress items (e.g., both rated 8 on days 1 and 5). Tallying the number of green cells and converting to a percentage yields a PCC of 30.77 (*c*-value < .001) for the pattern. This exact-match approach offers a distinct way of conceptualizing and testing causal hypotheses, differing from the analysis of concurrent changes over time.

## Ordinal Pattern Analysis

**Figure 6:**
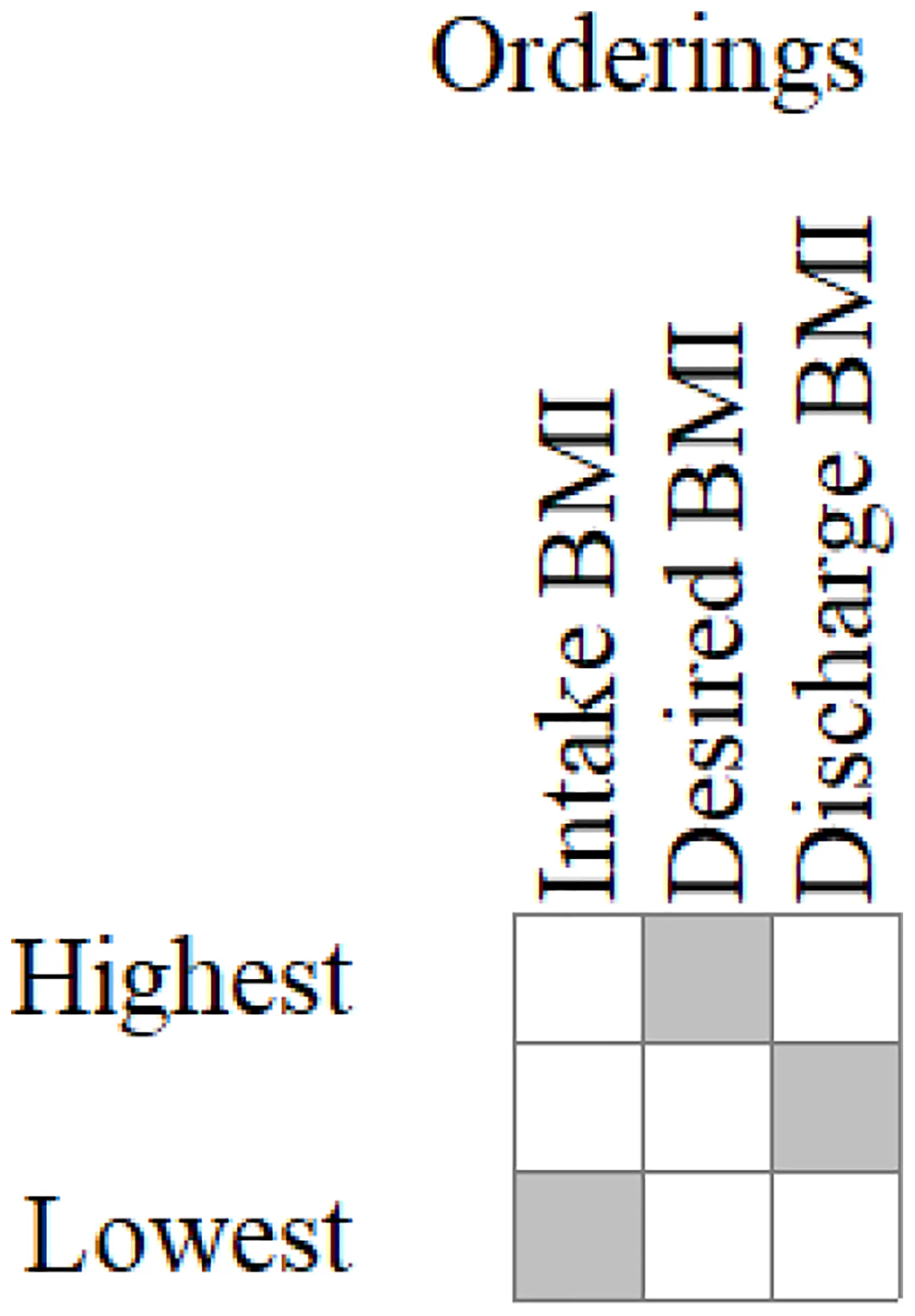
Predicted ordinal pattern of BMI values

Even relatively simple repeated-measures designs can be effectively used to gather data for explanatory, person-oriented purposes. For example, consider the data presented by Hand et al. (1994, p. 311) from twenty anorexic women. Upon admission to the hospital, each woman’s body mass index (BMI) was recorded along with her self-reported desired weight (converted to the BMI metric). BMI was measured again at discharge, resulting in three scores for each woman: Intake BMI, Desired BMI, and Discharge BMI. At first glance these data could be summarized in terms of average pre–post change or modeled using standard growth curve techniques. In the OOM software, however, an *Ordinal Pattern Analysis* (Beechey, 2023, Grice et al., 2015) can instead be used. Developed from the work of Thorngate and Carroll (1986), this procedure begins with a visual specification of the expected ordinal pattern across the repeated measures orderings. As can be seen in Figure 6, the expectation for this example is that each woman will desire a higher body weight (recorded as BMI) but will achieve a more modest outcome by the time of discharge. This outcome will nonetheless be an improvement in her BMI compared to intake.

The expected ordinal pattern in Figure 6 was derived in an entirely *ad hoc* fashion. Ideally, we would be working from an integrated, causal model like the one above in Figure 2. Such a model would be grounded in the lived experiences of anorexic women and would represent the mechanisms and processes believed to generate the observed outcomes. It would also include at least one final cause, most notably the woman’s desired body weight as described above. Recall that final causes represent natural end states or goals that direct human behavior. In this study, each woman’s self-reported desired body weight functions as a personal goal or end state she is pursuing. Setting a goal that is unrealistically high or excessively low could significantly influence her emotional experiences and engagement in therapy. Therefore, including each woman’s desired body weight as a final cause would be an important component of any comprehensive causal model of this study. For examples of causal, iconic models that incorporate final causes, see Grice (2015), Grice et al. (2017) and Grice and Jones (2024).

**Figure 7:**
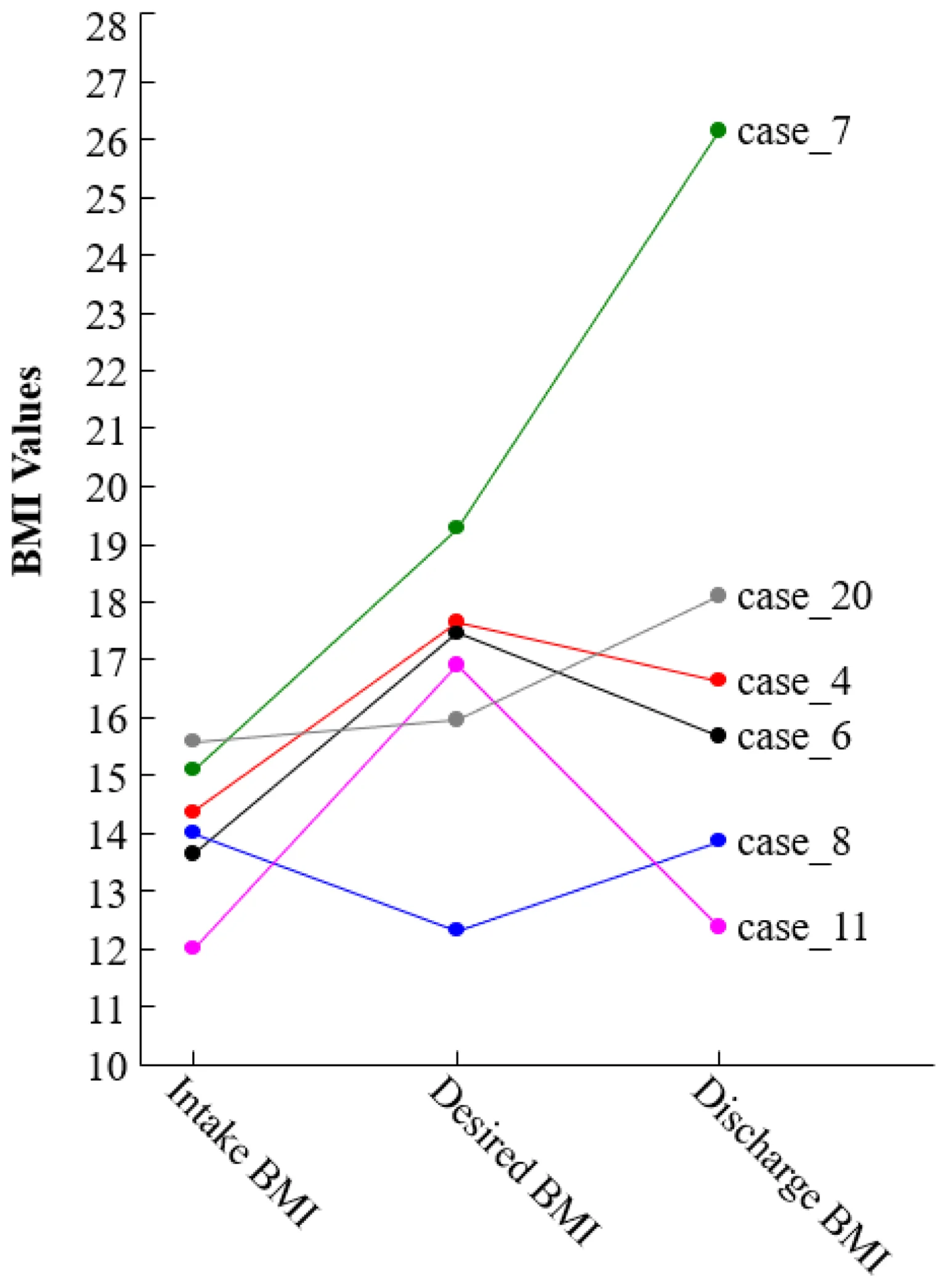
BMI scores for six anorexic women.

Without such a model the OOM analysis nonetheless proceeds by examining the pattern of BMI values for each woman. Figure 7 shows the results for six women, and as can be seen, they varied substantially in terms of their Intake, Desired, and Discharge BMI values. All twenty women could be shown in the graph, but it would be too cluttered to allow for the interpretation of individual women. The number of women whose three BMIs fit the ordinal pattern in Figure 6 are next tallied and reported as a Percent Correct Classifications (PCC) index. For these data only a slight minority of women (8 of 20) fit the ordinal pattern, PCC = 40.00%. With 60% of the women failing to match expectation, this result would likely be interpreted as offering weak evidence for the accuracy of the presumed explanatory model being evaluated even though the chance-value from the randomization test is low (*c*-value = .010). Moreover, of the twelve women who did not fit the pattern, nine demonstrated an increasing monotonic pattern across the three orderings (e.g., Case #7 in Figure 7). These nine women reported desired BMIs (converted from weights) that were higher than their intake values, and by discharge they had exceeded those expectations in BMI units. In other words, they underestimated their weight gains.

Pairwise comparisons with randomization tests for the ordinal pattern in Figure 6 can also be conducted in *the Ordinal Pattern Analysis*, the results of which are reported in Table 1. Of particular interest, 85% of the women had higher BMIs at discharge than at intake (*c*-value = .001). Thus, regardless of whether they overestimated their desired BMIs, a large majority increased their BMIs following therapeutic intervention. Only three women did not fit either the pattern in Figure 6 or the increasing monotonic pattern described above. One woman, somewhat unexpectedly, desired a lower body weight at intake, whereas two others desired higher body weights but had lower BMIs after therapy compared to intake. Further investigation of these three women – for example, through clinical interviews – could be particularly fruitful for refining a causal model underlying the therapeutic intervention. By eschewing means, variances, and traditional significance tests, OOM permits the researcher to focus on individuals in this manner.

**Table 1:** Pairwise Ordinal Pattern Analysis results

Comparison	*f_(op)_*	*f_(ccp)_*	*PCC*	*c*-value
Intake BMI < Desired BMI	20	19	95.00	< .001
Intake BMI < Discharge BMI	20	17	85.00	.001
Desired BMI > Discharge BMI	20	10	50.00	.585

*Note. f_(op)_* = frequency of observed pairs; *f_(ccp)_* = frequency of correctly classified observed pairs.

The *Ordinal Pattern Analysis* treats observed differences as either consistent with the predicted ordinal pattern (correct classifications) or inconsistent with it. Consequently, small differences in BMI values in the analyses above were treated as equivalent to large differences. An important option available in this analysis, and in most other procedures in the OOM software, is the ability to specify a *Classification Imprecision value*. Differences equal to or less than this value are treated as equivalent in the analysis being conducted. For example, suppose the doctors or therapists in the anorexia study considered changes of 3 BMI units or less to be clinically or theoretically inconsequential. Differences within this range would be treated as equivalent in the analysis and counted as incorrect classifications reducing the PCC for the Intake < Discharge BMI comparison from 85% to 40% (*c*-value = .100). By setting an imprecision value, then, researchers have some control over whether small differences are treated as meaningfully distinct from larger ones. Another approach for preserving distinctions between relatively small and large differences is to block the orderings and use strictly categorical (group-based) procedures available in the OOM software, as described below.

## Ordinal Patterns with a Grouping Ordering

Drawing an *inference to best explanation* requires at least two competing causal interpretations of the observed effects. For example, a wet lawn observed in the morning could be explained by an overnight rain, condensation, or the operation of a sprinkler system. In each example above, two competing explanations were described generically as *per se* causes (as described within a theoretical model) versus accidental causes (described as physical chance). Stronger explanatory inferences can be made when researchers directly pit one causal model against another, such as when comparing different groups across treatment conditions and/or a control group. Such designs typically include both a between-subjects grouping ordering (variable; e.g., treatment vs. control) and a repeated-measures ordering. For example, Hand et al. (1994, p. 229) report another study involving anorexic women that includes both types of orderings. Specifically, seventy-two women with anorexia were assigned to one of three conditions: Cognitive Behavioral Therapy (CBT), Family Therapy, or a Control group. Body weight, measured in pounds, was recorded both before (WghtPre) and after (WghtPost) treatment. Treatment condition served as the between-subjects ordering, and weight as the withinsubjects (repeated-measures) ordering.

Ideally, an OOM analysis of these data would begin with the presentation of integrated causal models for the Cognitive Behavioral and Family Therapy interventions. Such models would clearly explicate the mechanisms of change responsible for weight gain in women with anorexia. More sophisticated versions could even incorporate individual characteristics tailored to specific dispositions or circumstances known to impact therapeutic efficacy. In the absence of such integrated models, we can still proceed with the analysis and demonstrate how to draw an inference to best explanation by considering the two therapy conditions, the control group, and physical chance.

**Figure 8:**
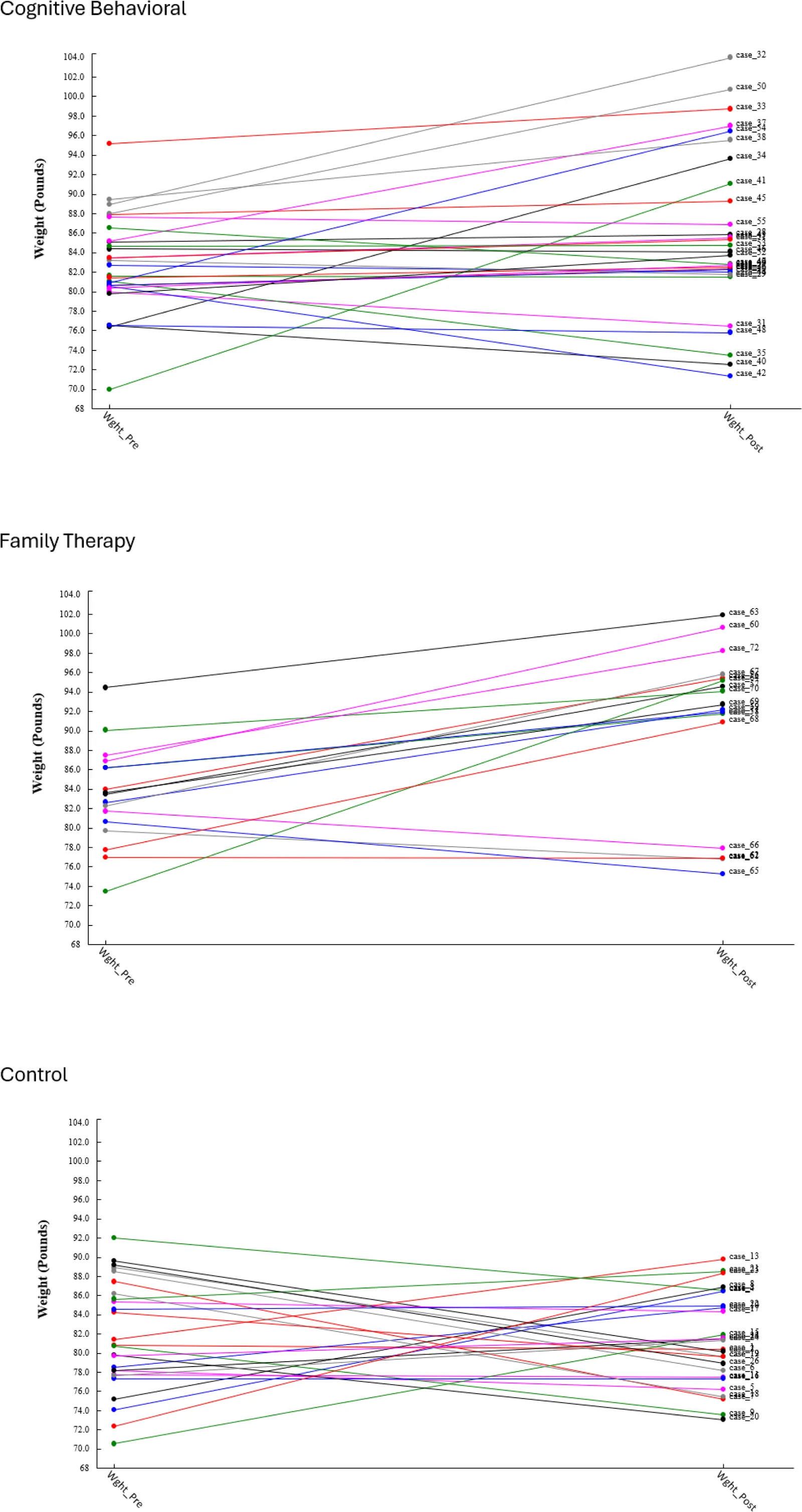
Visual representation of weight gain separated by group

The analysis begins by visually examining plots of the measured weights separated into the three groups. As can be seen in Figure 8, women in all three groups show variability in their pre- and post-treatment gains or losses. Some women gained weight while some women lost weight, and the magnitudes of differences varied substantially. Still, the “eye test” (Edwards et al., 1963) reveals no consistent gains or losses for women in the Control group, while an apparent majority of those in the Family Therapy group gained weight after the intervention. The *Ordinal Pattern Analysis* in the OOM software tallies the number of women whose pre- and post-treatment weights match the expected ordinal pattern; namely, WghtPost > WghtPre. Women whose weights match this pattern are considered correctly classified, and their percentage is reported as the PCC index. Each treatment group PCC can also be assessed via a randomization test, and the method chosen for these data randomly swaps the pre- and post-treatment weights for each woman for each of the 1000 iterations of the test. As can be seen in Table 2, and consistent with Figure 8, the Family Therapy treatment yielded the highest percentage of women who gained weight (a positive outcome), followed by the CBT group, and then the controls. The chance-values for the Family Therapy and CBT groups were low, particularly for the former group. Thus, results for each therapy were not plausibly attributable to physical chance alone. For descriptive purposes only, minimum, maximum, and median weight gains are also reported in Table 2. A median weight gain of 9 pounds for the Family Therapy group – with one woman gaining almost 22 pounds – is undoubtedly clinically relevant for these anorexic women.

**Table 2:** Comparisons of treatment effectiveness from the Ordinal Pattern Analysis

Treatment	*f_(o)_*	*f_(cc)_*	*PCC*	*c-value*	*Min*	*Max*	*Mdn*
Family Therapy	17	13	76.47	.03	-5.30	21.50	9.00
CBT	29	18	62.07	.15	-9.10	20.90	1.40
Control	26	11	42.31	.78	-12.20	15.90	-.35

*Note. f_(o)_* = observed frequency; *f_(cc)_* = frequency of correctly classified observations.

It is unfortunate that no other variables are recorded in the data set, as the next question for the OOM modeler would be, “Why did some of the women who underwent the interventions, particularly in the generally successful Family Therapy group, fail to gain weight?” The OOM software includes features that make it easy to identify and mark those women who, contrary to prediction, did not gain weight after therapy. Additional variables (orderings) could then be explored with the hope of finding more complex patterns that would increase the PCC indices and ideally lead to improvements in the causal models being tested. Do particular family dynamics hinder the effectiveness of Family Therapy? Do sensing personality types respond better to CBT than intuitive types? By focusing on patterns of observations and causal modeling, OOM encourages the exploration of such questions at the level of the individuals in the study.

Extending the explanatory inference to the between-subjects ordering, we can also compare the causal efficacy of the two therapies. The most effective therapy, supported by the more accurate causal explanation of weight gain, should produce change in a greater percentage of women than the other conditions. The *Ordinal Pattern Analysis* provides several options for making these group comparisons, with results presented in Table 3. As can be seen, ~34% more women in the Family Therapy group gained weight compared to women in the control group. The plausibility of this difference being due to physical chance was also low (*c*-value = .03). The difference in PCCs between the CBT and control groups was arguably clinically relevant as well (~20%), although the *c*-value (.15) was not as impressively low. The difference in PCCs between the two treatments was lowest with the highest *c*-value (.33). These results support the causal models underlying the therapies in two ways. First, the two therapies outperformed the control group which serves as a model for the operation of accidental causes in the lives of anorexic women. Second, they both performed better than physical chance according to the randomization tests, which simulated accidental causality from the data. The results do not provide strong support, however, for inferring (concluding) that the Family Therapy causal model is superior to the CBT model for explaining weight gain among women with anorexia. While results for the Family Therapy group appear superior to the CBT group in Figure 8, the similar PCCs and relatively high *c*-value make it difficult to infer that one model is meaningfully more accurate than the other. The findings may suggest that multiple causal pathways are producing weight gain in anorexic women, or that both therapies are operating through a shared underlying causal process. For example, so-called “common factors,” such as therapist rapport, client expectations, and general therapeutic support, may account for the weight gain observed in both the Family Therapy and CBT groups.

**Table 3:** Follow-up pairwise comparisons from the Ordinal Pattern Analysis

Comparison	*PCC*_1_	*PCC*_2_	*Diff*	*c-value*
FT_1_ vs. CBT_2_	76.47	62.07	14.40	.33
FT_1_ vs. Control_2_	76.47	42.31	34.16	.03
CBT_1_ vs. Control_2_	62.07	42.31	19.76	.15

## Proportional and Logical Patterns

As an example of how formal causes, which are concerned with structure, shape, configuration, and the essential organization of some thing or phenomenon, can be approached from the OOM perspective, consider data reported by Stemmler (2020, p. 7) regarding the effects of LSD on selfreported psychological states, particularly with regard to how they might mimic symptoms of psychoses. Sixty-five students took the drug under controlled conditions and were then assessed for three states using a binary (Yes/No) response format: Narrowed Consciousness, Thought Disturbance, and Affective Disturbance. Does LSD generate any one of these experiences or some combination thereof? Given what has been written above, the reader will likely notice that this question involves two types of causality. The first concerns efficient cause: Does taking LSD produce these particular symptoms after some period of time? The second concerns formal cause: Do the symptoms mimic the *form* of those experienced in psychosis (i.e., narrowed consciousness, thought disturbance, and affect disturbance), and do they occur in a specific combination (or configural *form*)?

A straightforward initial step in the OOM analysis of these data is to tally the number of students who endorsed each psychological state. Results showed that a majority of students reported experiencing each state: Narrowed Consciousness (NC, 57%), Thought Disturbance (TD, 52%), and Affective Disturbance (AD, 65%). Next, configurations of these states can be examined by crossing the three binary orderings and tallying the frequency of each resulting pattern. Table 4 shows the eight possible configurations along with their observed frequencies and proportions. As can be seen, the most frequent configuration (*n* = 20) was the simultaneous occurrence of all three states, expressed logically as NC_yes_^TD_yes_^AD_yes_, where ^ represents logical conjunction. By comparison, not a single student reported the simultaneous absence of all three states (NC_no_^TD_no_^AD_no_, *n* = 0). Using the *Single Ordering Proportion Analysis* in the OOM software, the observed proportions can be compared to theoretically expected proportions using randomization tests (see Grice, 2026, for details on how these tests are performed). For this analysis, equal expected proportions were specified for the eight configurations (.125, or 1/8, for each). As can be seen in Table 4, the proportional differences varied in magnitude, with two groups yielding notable absolute magnitudes. The NC_yes_^TD_yes_^AD_yes_ configuration was observed more often than expected (.308 vs. .125, *c*-value = .001), while the NC_no_^TD_no_^AD_no_ configuration occurred less frequently than expected (0 vs. .125, *c*-value = .031).

**Table 4:** Single Ordering Proportion Analysis results

Configuration	*f_(o)_*	*p(_o_)*	*p(_e_)*	*p(_o-e_)*	*c*-value
NC_no_^TD_no_^AD_no_	0	.000	.125	-.125	.031
NC_no_^TD_no_^AD_yes_	15	.231	.125	.106	.093
NC_no_^TD_yes_^AD_no_	10	.154	.125	.029	.683
NC_no_^TD_yes_^AD_yes_	3	.046	.125	-.079	.168
NC_yes_^TD_no_^AD_no_	12	.185	.125	.060	.371
NC_yes_^TD_no_^AD_yes_	4	.062	.125	-.063	.243
NC_yes_^TD_yes_^AD_no_	1	.015	.125	-.110	.053
NC_yes_^TD_yes_^AD_yes_	20	.308	.125	.183	.001

Note. *f_(o)_* = observed frequency, *p_(o)_* = observed proportion, and *p_(e)_* = expected proportion.

**Figure 9:**
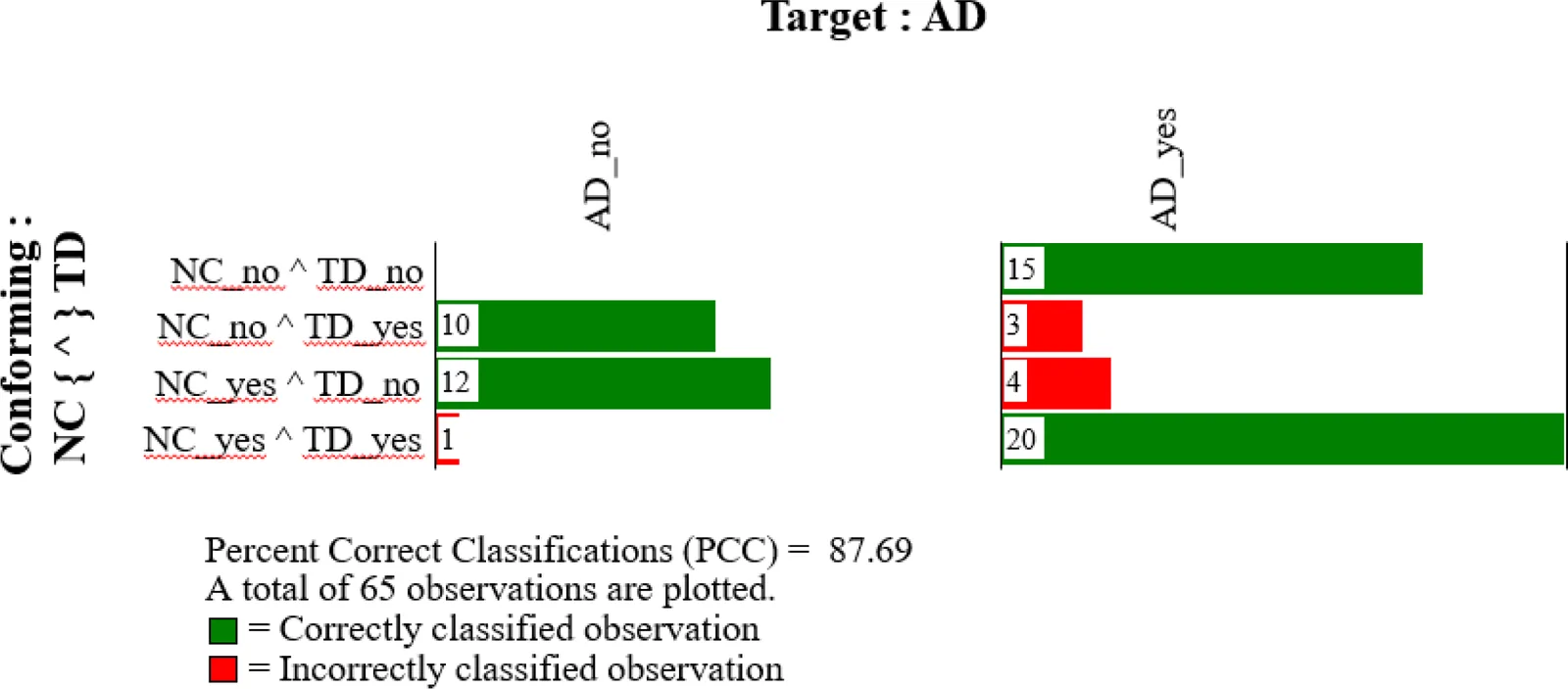
Multigram of configurations of experiences reported by students who consumed LSD

Going beyond this simple proportional analysis, the *Build/Test Model* procedure in the OOM software really brings the *form* of these results into sharp relief, rendering it both striking and visually compelling. In order to conduct the analysis, two of the orderings (variables) must be crossed, the choice of which is arbitrary. We crossed the Narrowed Consciousness and Thought Disturbance orderings and then rotated the resulting ordering to conformity with the Affective Disturbances ordering using a Procrustes rotation in the *Build/Test Model* procedure (see Grice, 2011). This rotation method is equivalent to a naïve Bayesian classifier in this instance (Sauer, 2018). The primary output from the procedure is the multi-unit frequency histogram, or “multigram”’ for short, shown in Figure 9. As can be seen, the frequency bars are color-coded; green for correctly classified and red for incorrectly classified. The correctly classified observations are summed and reported as the PCC, 87.69%, which is impressively high. Randomizing within the crossed ordering yields a low *c*-value (< .001) from the randomization test, thus supporting a causal (i.e., non-chance) interpretation of the results.

A more detailed examination of the multigram in Figure 9 reveals that the bars correspond to the configurations presented in Table 4. It is also now visibly clear that not a single student reported the simultaneous absence of all three states (NC_no_^TD_no_^AD_no_). The most frequent configuration was the simultaneous occurrence of all three states (NC_yes_^TD_yes_^AD_yes_, 30.8%), as noted above, followed by an Affective Disturbances only configuration (NC_no_^TD_no_^AD_yes_, 23.1%). Importantly, the multigram also reveals a third meaningful configuration which can be formed by combining the two groups with green bars under the “AD_no” column of the multigram (see Figure 9). This novel configuration is: Narrowed Consciousness or Thought Disturbance, without Affective Disturbances, expressed logically as ((NC_yes_ ⊕ TD_yes_)^AD_no_), where ⊕ represents exclusive disjunction (XOR). The highest percentage of students (*n* = 22, 33.8%) matched this configuration.

The results from the *Build/Test Model* procedure clearly indicate that the experiences reported by the students were not uniform. This means that, while LSD is working as an efficient cause in each person, it is also working in combination with other factors, producing three distinct patterns of subjective experience. Some students experienced only affective disturbance, others reported narrowed consciousness or thought disturbance without affective changes, and a third group experienced all three states simultaneously. These patterns likely arose from unique combinations of personality traits, biological predispositions, and personal histories interacting with LSD. Identifying the specific factors and how they combine into coherent wholes that result in the observed configurations amounts to discovering the formal causes at work. The discovery of the patterns thus marks the beginning of the more challenging phase of scientific inquiry. The ultimate goal is to develop an integrated causal model that accounts for the three observed configurations and the overall pattern of results. This is precisely the purpose of OOM: to enhance visual transparency, simplify analyses, and help researchers identify meaningful patterns and pursue deeper causal questions.

## Persons in Experimental Designs

Because they are rooted in the natures of things, causes are to be sought primarily within individuals rather than within aggregates. As an example of how traditional aggregate-based researchers using conventional experimental designs can benefit from this person-oriented principle, consider Van Dessel and Houwer’s (2019) study on modifying implicit and explicit attitudes. Implicit attitudes are generally more resistant to change than explicit attitudes because they involve the automatic activation of stable associations in memory. Van Dessel and Houwer tested whether hypnotic suggestion could facilitate deeper processing of counter-attitudinal information, thereby increasing the malleability of implicit attitudes. College students (*n* = 60) first read fictitious stories about two social groups (Niffites and Luupites) in conflict. For half the participants, the Niffites were portrayed positively and the Luupites negatively; for the other half, the descriptions were reversed. They were then assigned to either a hypnosis or relaxation condition, followed by exposure to either counter-attitudinal information or a control narrative. Participants in the hypnosis condition received a suggestion to process the upcoming information deeply, whereas those in the relaxation condition were simply instructed to relax. Participants in the counter-attitudinal condition read new descriptions of the Niffites and Luupites in which the valences of the groups were reversed such that the previously positive group was portrayed as hateful and aggressive, while the previously negative group was portrayed as noble and peaceful. Those in the control condition read a narrative about the fauna in each group’s residential area. Explicit attitudes about the groups were then measured using a four-item semantic differential scale, with scores ranging from -8 (extremely negative attitude toward the initially positive group) to +8 (extremely positive attitude toward the initially positive group). Implicit attitudes were assessed with the Implicit Association Test (IAT) and scored so that negative and positive scores indicated a relative dislike of or preference for, respectively, the initially positive group.

Using a 2x2 (Hypnosis/Relaxation x Counter-Attitudinal/Control) ANOVA Van Dessel and Houwer (2019) found that, in the aggregate, explicit attitudes toward the initially positive social group changed as expected for those who received counter-attitudinal information. Specifically, examination of marginal means indicated that students in the counter-attitudinal conditions reported negative attitudes (*M* = 2.63, *SD* = 2.58) toward the group while those in the control conditions reported positive attitudes toward the group (*M* = 5.05, SD = 2.15), *F*(1,56) = 176.17, *p* < .001, η^*2*^ = .76. Also as expected, a 2x2 ANOVA on implicit attitude scores revealed a significant interaction, *F*(1,56) = 5.39, *p* = .024, η^*2*^ = .09. Examination of the means showed that the Hypnosis/Counter-Attitudinal condition had the lowest mean preference for the positive-turned-negative group (*M* = .01, *SD* = .20) compared to the other three conditions (*M* = .17, *SD* = .22, for Relaxation/Counter-Attitudinal; *M* = .16, SD = .13, for Relaxation/Control; *M* = .22, *SD* = .19, for Hypnosis/Counter-attitudinal). The comparatively high means for these three conditions indicate a retained implicit preference for the initially favored social group regardless of whether they turned negative or stayed positive. In other words, in support of Van Dessel and Houwer’s hypothesis, the hypnosis resulted in a lower mean implicit attitude score for those who read the counter-attitudinal information for the initially positive group.

**Figure 10:**
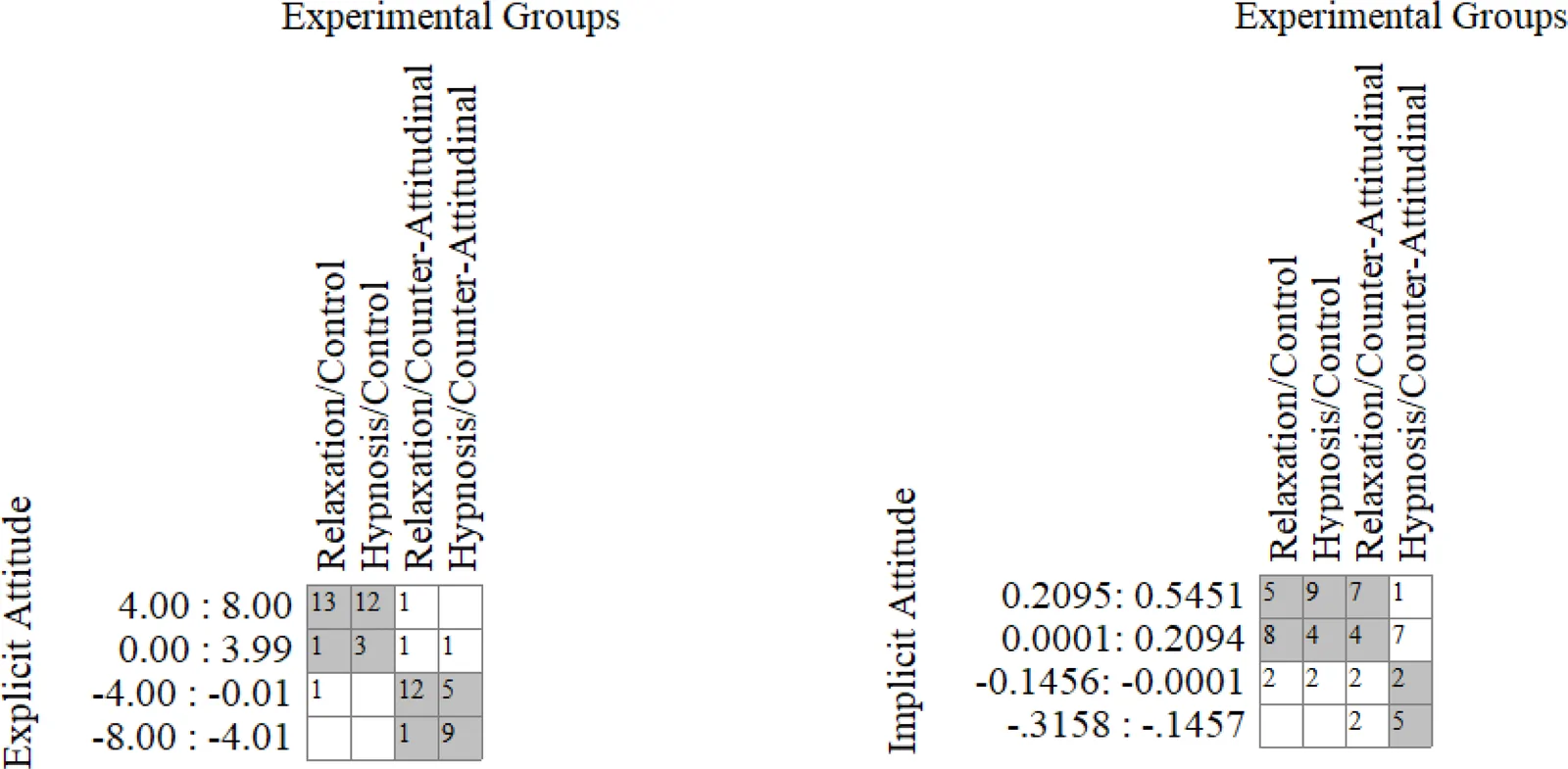
Results for Crossed Orderings Pattern Analyses of Explicit and Implicit Attitudes. *Note*. Grey cells indicate predicted patterns of results, and numbers within the cells indicate numbers of participants. For explicit attitudes (left image), positive ratings are expected to flip to negative ratings when counter-attitudinal information is presented. For implicit attitudes (right image), positive values are expected to flip to negative values only for the Hypnosis/Counter-Attitudinal group.

The clearest and most effective way to analyze these data in OOM, which will make the patterns both visible and theoretically meaningful, is to first bin the two dependent variables. Beginning with the explicit attitude ratings, four categories were created to represent different levels of attitude extremity: strongly positive (4.00–8.00), mildly positive (0.00–3.99), mildly negative (−0.01 to −4.00), and strongly negative (−4.01 to −8.00). Next, the two independent variables were crossed to form one ordering with four groups: Relaxation/Control, Hypnosis/Control, Relaxation/CounterAttitudinal, and Hypnosis/Counter-Attitudinal. These orderings (explicit attitude and experimental condition) were then analyzed using the *Crossed Orderings Pattern Analysis* in the OOM software. As shown in the left image of Figure 10, the analysis creates a 4×4 contingency table in which the user selects (shades) the cells that represent the theoretically expected pattern. The shaded cells in the figure are in this case analogous to the main effect in the ANOVA above, but based on individuals rather than means. Specifically, participants in the control conditions are expected to maintain positive attitudes toward the initially favored group, while those in the counter-attitudinal conditions are expected to shift to negative attitudes. Of the 60 participants, 56 fell into the expected (shaded) cells, yielding a PCC index of 93.33%. Notably, 27 of the 30 individuals who received counter-attitudinal information shifted their explicit attitudes from positive to negative. A randomization test, in which attitude scores and experimental group membership were randomly shuffled, produced a very low *c*-value (< .001), indicating that the observed pattern is not plausibly explainable by physical chance alone. In sum, these results strongly support the researchers’ theoretical explanation for explicit attitude change at the level of the persons in the study.

Turning to the implicit attitude scores from the IAT, these were similarly binned into four categories: strongly positive (.2095 to .5451), mildly positive (.0001 to .2094), mildly negative (−.1456 to −.0001), and strongly negative (−.3158 to −.1457). These bins were created based on the observed range of values and in a manner to preserve the importance of their signs; specifically, negative values indicate a dislike for the initially positive group whereas positive scores indicate a preference for the group. This implicit attitude ordering was then crossed with the experimental condition ordering using the *Crossed Orderings Pattern Analysis*. As shown in the right image of Figure 10, the shaded cells represent Van Dessel and Houwer’s (2019) prediction that only participants in the Hypnosis/Counter-Attitudinal condition would shift to negative implicit attitudes toward the initially positive social group, while the other three conditions were expected to maintain positive implicit attitudes. Of the 60 participants, 44 fell into the expected (shaded) cells, yielding a PCC of 73.33% (c < .001). Examination of the frequencies revealed that a majority of individuals in the Relaxation/Control (87%), Hypnosis/Control (87%), and Relaxation/Counter-Attitudinal (73%) conditions maintained positive implicit attitudes, as expected. However, in the Counterattitudinal/Hypnosis condition, only 7 of the 15 participants (47%) showed the expected negative implicit attitudes, while 8 maintained positive attitudes. In other words, contrary to prediction, most participants in this condition did not change their implicit attitudes. Van Dessel and Houwer’s (2019) conclusion that they “…observed rapid change in implicit evaluations as the result of a single piece of counterattitudinal information” and that “hypnotic suggestions of enhanced processing of the counterattitudinal information moderated this effect” (p. 1367) is therefore in need of nuance. Not everyone in the Hypnosis/Counter-Attitudinal condition produced IAT values that indicated a shift to a negative implicit attitude to the positive-turned-negative social group. In fact, a slight majority showed an implicit preference for this group.

A number of interesting person-oriented questions arise from this more accurate view of the responses of the participants: Was the hypnosis ineffective for the eight individuals who did not match the expected pattern in the Hypnosis/Counter-Attitudinal condition? Do these individuals share particular features, such as gender, lower initial explicit attitudes, or older age? Were they unconvinced of the counter-attitudinal information? We in fact conducted a number of exploratory analyses but failed to identify any clear distinguishing characteristics between these eight individuals and the seven who did match the predicted pattern.

## Discussion

The diverse examples above demonstrate that Observation Oriented Modeling (OOM) entails a Gestalt shift away from the analysis of means and variances and the estimation of abstract population parameters toward the detection, analysis, and causal explanation of patterns of observations at the level of concrete persons. The examples and methods presented above also make clear that placing pattern detection and explanation at the center of scientific inquiry entails far more than a simple modification of existing methods of data analysis. Rather, it reflects a broader reorientation of perspective that involves philosophy, scientific inference, generalizability, and statistical methodology. We believe that such a reorientation benefits not only person-oriented researchers but psychologists in general, and in what follows we discuss its key features.

### Philosophy

It is well known that psychology’s experimental methods and statistical tools of data analysis are rooted in Logical Positivism, a philosophical movement that was largely abandoned in the mid-20th century. Costa and Shimp (2011) describe why this history matters for us today:

It matters because a commitment to a philosophy of science generally colors what one believes science is, and that in turn impacts what one believes is good science, what is appropriate scientific methodology, and what is the proper relation between science, truth, and society (Feist, 2006; Fuller, 2003; Koch, 1992a, 1992b; Koch & Leary, 1992; Lacey, 2005; Longino, 1990; Ravetz, 1971). Therefore, it affects the process of doing science… (p.35)

They also point out that positivism regards methodology as independent of and superior to theory in scientific endeavors. The premise was that methods alone could yield objective truth untainted by theory, which could be too easily colored by subjective whims. Importantly, logical positivism also posits a largely subjective or psychological view of causation. Causes are not objective powers in nature, nor are they necessarily connected to their effects. Instead, causation is reduced to the constant conjunction of events in our experience, coupled with our psychological habit of expecting one event to follow the other. In other words, if event B regularly follows event A in time, and we come to expect B whenever we see A, the positivist would refer to A as the cause and to B as the effect. Modern psychologists’ mantra that “correlation does not imply causation” because observed associations may reflect confounding variables or other noncausal relationships remains consistent with this broader positivist view. Causal claims must still be supported by empirical regularities rather than by directly apprehending necessary causal connections, which is why psychologists generally regard randomized controlled experiments as the strongest basis for causal inference. Random assignment to conditions is intended to eliminate alternative explanations, allowing the remaining association between treatment and outcome to be interpreted as evidence of a causal relationship. Nevertheless, the underlying conception of causation remains fundamentally associational, with causal inference resting on observed covariation rather than on an understanding of the objective causal powers responsible for the observed effects.

As our analyses and discussions of the example studies above make clear, psychology need not remain in the quagmire of logical positivism. By embracing Aristotelian philosophy, or some form of realism derived from it, the field can move forward (Arocha, 2021; Chirkov & Anderson, 2018a, 2018b). The iconic model in Figure 2 and the various discussions of causality surrounding the studies above vividly illustrate the superior nuance, clarity, and sophistication that Aristotle’s comprehensive understanding of causality offers scientific investigation. In fact, for Aristotle, scientific knowledge is acquired when the causal structure of a natural system has been uncovered and formalized in thought. Causes are moreover understood as powers (or capabilities) rooted in the natures of things (see Mumford and Anjum’s, 2011, *causal powers* thesis). It is through the exercise of these powers that organisms, for instance, realize their characteristic ends. Honeybees fly, forage, and construct honeycombs; rattlesnakes slither, capture prey, and dwell in burrows; and amoebae move about, engulf nutrients, and reproduce through cell division. In each case, the characteristic activities of the organism arise from its nature and express the causal powers proper to the kind of thing it is. Wallace’s (1996) *The Modeling of Nature* is a *tour de force* discussing causality from this perspective, demonstrating how iconic models can be constructed for organisms ranging from the simplest to the most complex, and showing how causal powers may be hierarchically organized. Wallace adopts the Moderate Realism found in the Aristotelian-Thomistic synthesis (Wallace, 1983) and demonstrates how it leaves room for a science concerned with both quality and quantity; that is, for a science that is not regarded as solely a quantitative endeavor.

To embrace this greater sophistication, psychologists’ methods of model building must not be restricted solely to mathematical models nor to simple diagrams consisting of nodes, boxes, or ellipses connected with arrows (like directed acyclic graphs, path models, or structural equation models). Instead, iconic models like the integrated model in Figure 2 should be explored and utilized. Such models are person-centered, can incorporate different causes, can include qualitative and quantitative features, and can clearly differentiate between distinct psychological elements such as emotions, thoughts, and memories. They are inspired by the complexity one finds in modern biochemical models, such as the model of the Kreb’s cycle. The model in Figure 2 is admittedly simple compared to most biochemical models, but if taken seriously, it can nonetheless serve as the starting point for a more sophisticated and complete understanding of how Thorne and Kamps’ (2008) behavioral intervention works. Are we to understand the learning that takes place in the 12 students, for example, as purely mechanical in the tradition of operant conditioning and efficient-cause-only S-R models, or are we to include final causes as shown in our integrated model?

Rychlak’s *Logical Learning Theory* (1988; for example, see Rychlak’s comparison between a *response* and a *telosponse*) and Powers’ (1973) *Perceptual Control Theory* have been shown to be viable frameworks for thinking about behavioral change in more sophisticated terms that are consistent with the Aristotelian-Thomistic causal perspective underlying OOM and the integrated model in Figure 2. In any case, efforts to make explicit the underlying causes of the intervention program should, in turn, yield practical benefits for implementing and improving the program. Contrary to the dictates of logical positivism, then, realist philosophy regards theory construction and model building as superordinate to methodology in the quest for scientific knowledge. In brief, this shift in perspective will ensure that psychologists will not allow their methods to become their metaphysics (see Rychlak, 1985,1988).

### Scientific Inference

Modern data analysis in psychology is primarily inductive, as evidenced by the widespread reliance on various forms of significance testing throughout the discipline. Null hypothesis significance testing (i.e., the common *p*-value), re-sampling techniques, or Bayesian statistics are routinely used to estimate population parameters. A psychologist draws a specific sample and then computes a statistic which is treated as an estimate of the corresponding parameter for a general (and often hypothetical) population. A binary conclusion is then drawn and stated in simple language; for example, “the result was significant,” “the two variables were associated,” or the “the drug was effective.” Though routinely omitted, such statements should also include references to the population; for example, “the two variables are inferred to be associated *in the population*” or “the drug is inferred to be effective *at the level of the population*.” These more precise statements make it clear that the inference being drawn is inductive, moving from the specific sample to the general population. For the OOM analyst, however, the inference to be routinely sought from analyses is not inductive but either *abductive* or to *best explanation*, as demonstrated and discussed in the example studies above.

Consistent with Aristotelian philosophy, C. S. Peirce (1931-1958) regarded *abduction* as a third fundamental mode of reasoning, alongside induction and deduction (see also, Rozeboom, 1997). He considered it central to science as it entails reasoning from observed effects back to the most plausible unseen causes. For instance, observing wet grass, one may plausibly infer that it rained; seeing a frown on a friend’s face, one may infer having said something disagreeable; or hearing a crash in another room, one may infer that the cat knocked something over. In each case, the person moves from an observed effect back to a plausible but unseen cause. When multiple hypotheses are possible, selecting the most plausible one constitutes *an inference to best explanation*. For discussions of plausibility and inference to best explanation, see Campos (2011), and Lipton (2004), respectively.

Grice et al. (2017) provide an empirical example of perhaps the strongest way to draw an inference to best explanation. They created two competing integrated models and evaluated them using the same data set. By comparing the resulting PCCs, one model was judged to provide a more plausible explanation of the observed pattern than the other.

The study described above comparing the effectiveness of Family Therapy (FT) and Cognitive Behavioral Therapy (CBT) in promoting weight gain among anorexic women illustrates a different route to drawing an inference to best explanation. Recall the percentage of women who gained weight was determined for each therapy group and compared with the corresponding PCC obtained for a control group. The control group served as a benchmark, reflecting the influence of unspecified causes (*per se* and accidental) that might contribute to weight gain or loss in the absence of therapeutic intervention. In contrast, the FT and CBT groups reflected the operation of theoretically specified causes embodied in the respective treatment programs. The critical question, therefore, was whether the causal processes enacted by FT and CBT could outperform the unspecified causes represented by the control condition. The substantially higher PCCs observed for the FT (76%) and CBT (62%) groups suggested that they could (42% for controls). Moreover, the low chance values associated with these differences indicated that the observed results were not plausibly attributable to the unspecified causes represented by the control group alone. The best explanation for weight gain in each group was therefore inferred to be the respective causal model underlying that therapeutic intervention. However, because the PCCs for the two therapy groups were similar and the *c*-value from the randomization test was high (.33), neither putative causal model could be confidently identified as the better explanation for weight gain among anorexic women.

In the absence of competing causal models or comparison groups, an inference to best explanation can still be drawn provided that the researcher is able to offer a causal model capable of explaining the observed pattern of results. As demonstrated above, a randomization test can be used to evaluate the observed pattern against patterns generated by chance. By randomly shuffling the data, we simulate accidental causes, or physical chance, and thereby create a competing explanation for the observed pattern. If the observed PCC can be easily equaled or exceeded by PCCs obtained from randomized versions of the data, this suggests that the observed pattern could plausibly have arisen through accidental causes alone. Relative to situations involving two competing integrated models or comparison groups representing different causal models, this use of the *c*-value represents a comparatively modest basis for evaluating explanatory plausibility. Nevertheless, the *c*-value can serve a useful role provided researchers recognize that it constitutes only one piece of evidence among many. It should never be treated as the primary statistic yielded by an analysis, nor should it be interpreted using arbitrary cut-points (e.g., *c* ≤ .05). Rather, researchers should evaluate explanatory plausibility by considering the clarity and meaningfulness of the observed pattern, the Percent Correct Classification (PCC), the *c*-value, and broader theoretical considerations such as simplicity, coherence, scope, and explanatory power (Haig, 2005, 2014).

Although this abductive process of inference may appear more complex and uncertain than simply declaring a result “statistically significant,” it more accurately reflects the iterative, theory-laden, and fallible nature of scientific inquiry (Hubbard et al., 2019; Rozeboom, 1997). It is also consistent with recommendations offered in a special issue of *The American Statistician* (see Wasserstein et al., 2019), in which researchers were encouraged to abandon the language of “statistical significance” altogether and avoid the simplistic binary thinking often associated with traditional significance testing. Instead, Wasserstein et al. recommended reporting exact *p*-values while placing greater emphasis on effect sizes and the substantive meaning of results. In conventional analyses, this often entails focusing on quantities such as mean differences, correlation coefficients, and odds ratios. In the OOM analyses presented above, a similar principle was followed. Rather than fixating on *c*-values and arbitrary cutpoints, primary attention was directed first to the meaning of the observed patterns of results and second to the PCCs. The *c*-value was treated as supplementary information that aided in evaluating the plausibility of *per se* versus accidental causation. In this way, statistical analysis remains subordinate to the larger scientific task of identifying and evaluating the most plausible causal explanation of the observed pattern.

### Scientific Generalizability

A common question posed to person-oriented researchers is: “How do you generalize beyond the individuals in your study?” The question becomes especially pressing in singlesubject and small-*n* designs. From the OOM perspective, the answer is straightforward: generalization occurs through theory and exact replication. Once the causal structure of a natural system has been adequately mapped, scientists may reasonably expect the same explanatory model to apply to equivalent systems operating under similar conditions across different times and places. This expectation is consistent with the Aristotelian-Thomistic realist view that nature is orderly, lawful, and intelligible.

Consider again the behavior modification example above. The iconic model in Figure 2 specifies how the teacher may instill a sequence of internal causal states in students that culminate in productive behavior. To the extent that the relevant causal mechanisms can be instilled in students across classrooms, schools, cultures, and historical settings, the same intervention should tend to produce similar outcomes. Of course, the only way to test the validity of this expectation is to in fact conduct a series of exact replications across various places and with different students. The *c*-value, like *p*-values obtained from traditional significance tests, is unrelated to the probability that a finding will replicate. In other words, a low *c*-value does not imply that a result is either more or less likely to be reproduced in a subsequent study. For example, Grice et al. (2017, p. 16) reported a low *c*-value (.001) and a high PCC (72.73%) for a pattern of observations involving factors that influence men’s willingness to forgive. Despite these strong results, the pattern failed to replicate in a subsequent exact replication study (Jones et al., 2022). This example illustrates an important point: neither *c*-values nor *p*-values provide information about the likelihood of replication. In short, replicability remains one of the hallmarks of scientific knowledge, as it provides the primary basis for establishing the generalizability of a finding across persons, settings, and times, and there is no statistical shortcut to achieving it.

Beyond replicability, person-oriented researchers are familiar with another problem concerning scientific generalizability; namely, the aggregate-to-person generalizability problem which is more commonly referred to as the *ecological fallacy*. In more colorful language, Lamiell (2013) refers to the belief that average results provide accurate descriptions of individuals as *statisticism*, while Molenaar (2004) discusses the problem as one of mistakenly presuming *ergodicity* for *nonergodic systems* (see also, Richter’s, 2021, *psychological homogeneity assumption*). As illustrated by the experimental study on the stability of implicit attitudes above, the fact that a result based on group means was statistically significant does not necessarily imply that most individuals behaved in a manner consistent with the hypothesis (see Speelman & McGann, 2026). In fact, the OOM analyses revealed that a slight majority of participants in the Hypnosis/Counter-Attitudinal condition in the study actually behaved contrary to expectation. McManus et al. (2023) recently demonstrated that such contra-indicatory findings are not uncommon in social psychology. They further reported that a majority of psychologists surveyed believed that a statistically significant result implies that most individuals in a study behaved in accordance with the hypothesis (see also, Speelman, et al. 2024). Yet this inference does not follow from the logic of null-hypothesis significance testing. It represents another widespread misunderstanding attached to the *p*-value, akin to the mistaken beliefs that a low *p*-value indicates either a high probability of replication or a low probability of the null hypothesis being true (see Gigerenzer, 2004; Lambdin, 2011; Oakes, 1986). As with replication, the ecological fallacy is ultimately an empirical matter. Determining whether most individuals behave in a manner consistent with a theory or causal model requires analyzing the data at the level of the person. As demonstrated with the diverse examples above, OOM provides a direct, visually transparent, and readily interpretable framework for making this assessment.

Finally, a pragmatic response to the problems of generalizability and the ecological fallacy can be found in the growing interest in *N*-of-1 and other single-case designs within medicine and the health sciences (Guyatt, et al., 1986; Mirza, et al. 2017). Researchers employing these approaches begin by evaluating whether a treatment is efficacious for a particular individual rather than inferring efficacy from aggregate results. In this way, evidence is first established at the level of the person, where causal effects are directly experienced and observed. As findings accumulate across many individual cases, researchers can then examine the extent to which treatment effects recur across persons and contexts. Such aggregation makes it possible to assess the broader applicability of an intervention, compare its effectiveness with alternative treatments, and identify factors that may influence treatment outcomes. In turn, these findings can be used to refine explanatory models and improve understanding of the causal mechanisms underlying treatment efficacy.

### Statistical Methodology

Statistical methods have become increasingly complex and sophisticated in recent decades. Multilevel modeling, structural equation modeling, machine learning and predictive analytics, and agent-based modeling, to name just a few, now provide researchers with an extraordinary array of tools for analyzing data. Although this methodological evolution toward greater computational sophistication has been impressive, OOM represents a movement in the opposite direction. As with the tendency to place method before theory discussed above, an emphasis on ever more complex analytic techniques risks diverting attention from theory development and explanatory understanding. Increased computational complexity does not necessarily yield greater explanatory clarity, particularly at the level of the individual person.

In his paper *Statistical Models and Shoe Leather,* the influential statistician David Freedman questioned researchers’ growing reliance on, and fascination with, increasingly complex statistical methods. As summarized by Mason (1991), Freedman argued that:

Simple [statistical/data analytic] tools should be used extensively. More complex tools should be used rarely, if at all. Thus, we should be doing more graphical analyses and computing fewer regressions, correlations, survival models, structural equation models, and so on. (p. 338)

Freedman’s use of the phrase “shoe leather” served as a metaphor for the idea that researchers should spend more time reasoning about the question at hand and gathering the observations needed to answer it, much like a detective walking the streets (spending shoe leather) in search of clues to solve a crime. In other words, researchers should adopt the attitude of Sherlock Holmes trying to solve a ‘who dunnit’ mystery about causes rather than Sir Ronald Fisher partitioning variability in a data set. In this particular regard, it is telling that none of the OOM analyses presented above relied on the computation of a variance, or, for that matter, a mean or standard deviation. This fact underscores the extent to which OOM truly represents a Gestalt shift in perspective.

Compared to most contemporary statistical methods, then, OOM is remarkably simple. When specifically interacting with the software one does not rely on equations but rather on patterns and visual representations of observed versus predicted outcomes. The analyses typically proceed by directly comparing individual observations to theoretically specified patterns, rather than building complex mathematical models with multiple assumptions and parameters. Consequently, many of the technical concerns that accompany traditional statistical analyses, such as checking distributional assumptions, identifying problematic cases, or adjusting for Type I inflation, are largely bypassed. The study of weight gain among anorexic women undergoing Family Therapy or Cognitive Behavioral Therapy above is a particular demonstration of the efficiency of the OOM approach. Recall that pre- and post-intervention weights were recorded and analyzed for seventy-two women. A traditional approach to these data would require the researcher to address several statistical challenges faced when analyzing change scores across groups, including regression to the mean, baseline imbalances, low reliability of difference scores, and reduced statistical power. These issues frequently lead researchers to prefer ANCOVA over split-plot ANOVA (Vickers & Altman, 2001). By contrast, OOM simplifies the analysis by focusing on categorical patterns of change (improved vs. not improved) rather than on means and variances. Without means there can be no regression toward the mean, and so this problem is avoided as is the need to adjust for baseline differences. One caveat is that even small changes are classified as improvements or decrements; however, as noted earlier, the imprecision index in the *Ordinal Pattern Analysis* can be used to treat trivial differences as effectively zero. Furthermore, because OOM relies on randomization tests as aids for drawing explanatory inferences, it sidesteps concerns regarding statistical power as well as Type I, II, and III error rates (Harris, 1996). As discussed at length above, the logic and language of traditional hypothesis testing is eschewed, so there can be no sense in computing, for example, the probability of detecting a statistically significant result as is done in routine power computations.

One of the most important assumptions underlying traditional statistical analyses rooted in parameter estimation is random sampling (Berk & Freeman, 2003). Although this assumption is rarely acknowledged, the validity of population parameter estimates and the probabilities computed from convenience samples is highly questionable, regardless of whether they are derived from NHST, Bayesian, or resampling techniques. As Dugard (2014) notes, “Random sampling from a large, well-defined population or universe is a formal requirement for the usual interpretation of all commonly used parametric and nonparametric statistical tests” (p. 65). Kirk (1999) likewise emphasizes the critical role of random sampling: “Inferential statistics are used in reasoning from a sample to the population …any sampling method based on haphazard or purposeless choices such as enlisting volunteers, students enrolled in a psychology course, or every 10th name in an alphabetical list is called nonrandom sampling. The resulting samples, unlike random samples, don’t provide a sound basis for determining the properties of populations” (p. 248).

By employing randomization tests that can be adapted to any type of data (qualitative or quantitative), pattern among variables, or study design, OOM largely avoids this problem because such tests do not require random sampling (Manly, 2006, 2013). Within the OOM framework, the analyst need only address the assumption of exchangeability underlying the randomization test, which amounts to defending the conceptual appropriateness of the manner in which the data are randomly shuffled. Given that psychologists rely overwhelmingly on convenience samples, OOM is more consistent with prevailing research practices. It may also tacitly suggest that psychologists are often more interested in drawing explanatory inferences than in making inductive inferences about population parameters.

## Conclusion

In sum, OOM is a person-centered approach to data analysis and scientific inquiry that requires a positivist-to-realist Gestalt shift and a movement beyond traditional methods. The preceding studies and examples illustrate the central features and benefits of OOM associated with this shift. These include a focus on pattern detection at the level of the individual, evaluation of explanatory plausibility, and the construction of causal models that are representative of human nature. By streamlining statistical methodology, OOM helps researchers avoid many of the common errors associated with conventional statistical analyses (Hubbard, 2016; Lamiell, 2019; Speelman & McGann, 2026) while substantially reducing concerns about assumptions and technical minutiae. In doing so, it allows researchers to confront more directly the fundamental conceptual and methodological challenges facing psychology (Uher, et al., 2025). More practically, OOM enables person-oriented researchers to devote greater time and cognitive energy to theory development, causal modeling, and substantive interpretation. Ultimately, the value of OOM lies not merely in offering an alternative method of data analysis, but in reorienting psychological inquiry toward the discovery and explanation of meaningful patterns in human behavior

## Data Availability

Data sets analyzed in this paper are available upon request from the first author. The OOM software can be freely downloaded at http://www.idiogrid.com/OOM/

## Action editor

Lars-Gunnar Lundh served as action editor for this article.

## Open access

This article is distributed under the terms of the Creative Commons Attribution 4.0 International License (http://creativecommons.org/licenses/by/4.0/), which permits unrestricted use, distribution, and reproduction in any medium, provided you give appropriate credit to the original author(s) and the source, provide a link to the Creative Commons license, and indicate if changes were made.
